# The impact of China’s National Drug Centralized Procurement Policy on pharmaceutical firm innovation: evidence from a staggered difference-in-differences analysis

**DOI:** 10.1080/20523211.2026.2680235

**Published:** 2026-06-08

**Authors:** Ying Chen, Wen Feng

**Affiliations:** a Department of Health Policy and Management, School of Public Health, Peking University Health Science Center, Beijing, People’s Republic of China; b Key Laboratory of Health System Reform and Governance, National Health Commission of China (Peking University), Beijing, People’s Republic of China

**Keywords:** China’s National Drug Centralized Procurement Policy, pharmaceutical firm, innovation, staggered DID model, mediation analyses, heterogeneity analyses

## Abstract

**Background:**

China's National Drug Centralized Procurement Policy (NDCPP) aims to reduce drug prices through a ‘price-for-volume’ mechanism. While effective in cost containment, its impact on pharmaceutical firms’ innovation remains debated, with existing studies often focusing on short-term or static effects.

**Methods:**

This study utilises an unbalanced panel of 160 listed Chinese pharmaceutical firms from 2015 to 2023. We employ a multi-period staggered difference-in-differences (DID) model to identify the causal impact of the NDCPP on innovation input (R&D investment intensity) and output (authorised invention patents). Mediation models are constructed to test the roles of cost and operational efficiency.

**Results:**

Baseline estimates indicate that the NDCPP significantly increases innovation activities. The implementation of the NDCPP led to an increase in innovation input by 0.0118 units (representing a 1.18 percentage point increase in R&D intensity, or a 22.06% increase relative to the mean) and innovation output by 0.847 units (representing an average annual increase of 0.847 authorised invention patents per firm, or a 14.20% increase relative to the mean). Dynamic analyses reveal an immediate response and continuous enhancement, with promotional effects strengthening from the first to fourth years after policy implementation respectively. Mediation analyses show that cost efficiency significantly mediates innovation output, while strategic inventory management mediates R&D investment. Heterogeneity analyses demonstrate strong responses among private, large, and chemical pharmaceutical firms, with traditional Chinese medicine (TCM) firms also showing significant gains in innovation input.

**Conclusion:**

The result indicates a strategic reorientation of pharmaceutical firms, incentivizing a resource reallocation from marketing activities toward substantive R&D activities. The ‘price-for-volume’ effect offsets price deductions through rapid reorganisation of resources. Future policies should consolidate this momentum and guide enterprises toward high-quality, sustainable innovation.

## 1. Background

Over the past three decades, China’s pharmaceutical procurement system has undergone profound institutional transformation. Since the establishment of the Henan Pharmaceutical and Medical Device Procurement Consulting Center in 1993, centralised drug procurement has evolved through two major phases: an early stage without guaranteed purchase volumes and a more recent stage characterised by volume-based procurement. Following the enactment of the Tendering and Bidding Law in 1999 and the issuance of the Guiding Opinions on the Reform of Urban Medical and Health Systems in 2000, drug centralised procurement entered an exploratory phase marked by decentralised, province- or city-level initiatives (Li et al., 2025). Despite multiple procurement models, these early arrangements yielded limited effectiveness. Local governments largely acted as intermediaries, while actual purchasing power remained with medical institutions, leading to post-bid renegotiation between hospitals and manufacturers and leaving substantial room for rent-seeking behaviour (Hu & Mossialos, 2016; Zhu et al., 2023). Moreover, the separation of bidding from purchasing and the decoupling of price and quantity often undermined contract enforcement, resulting in the phenomenon of ‘winning the bid but losing the market’ (Wang et al., 2023).

Against this background, China launched the landmark National Drug Centralized Procurement Policy in 2018, beginning with the ‘4 + 7’ pilot program across four municipalities and seven sub-provincial cities (Sunshine Medical Procurement All-in-one, 2018). This reform replaced administrative pricing and secondary bargaining with volume-based centralised procurement through multi-party negotiation. Empirical evidence shows that the policy substantially reduced drug prices while significantly increasing procurement volumes of winning products, generating large cost savings for the healthcare system (Wang et al., 2023). However, the sharp decline in drug prices has also raised concerns about potential adverse effects on pharmaceutical firms’ innovation incentives due to the largely increased buyer power (Köhler & Rammer, 2012). In a sector characterised by long development cycles, high R&D costs, and substantial uncertainty (Zhang & Ren, 2022), whether firms remain willing and able to invest in innovation under intensified price competition has become a central policy and academic question.

Innovation capacity is the cornerstone of pharmaceutical firms’ long-term competitiveness, enabling them to secure key patents, establish technological barriers, and sustain growth in an increasingly complex market environment (Robb et al., 2022). Compared with most manufacturing industries, pharmaceutical innovation involves longer timelines, higher sunk costs, and greater failure risks (Zhang & Ren, 2022). Recent evidence suggests that developing a new drug requires, on average, more than ten years and USD 879.3 million when accounting for failures, based on U.S. market data from the Department of Health and Human Services (Sertkaya & Berger, 2024). And approximately 90% of candidate drugs fail during clinical trials (Shen, 2025), reflecting the high-risk and capital-intensive nature of pharmaceutical R&D on a global scale. Although the R&D costs for domestic Chinese pharmaceutical firms are generally reported to be lower, they are rapidly rising as the industry shifts toward original innovation. These features make pharmaceutical innovation particularly sensitive to policy-induced changes in prices, revenues, and market expectations.

From a theoretical perspective, volume-based procurement reshapes the external innovation environment through the joint interaction of price and quantity mechanisms. In the short term, sharp price reductions and heightened uncertainty may compress profit margins and crowd out high-risk R&D investment (Bloom, 2007). At the same time, policy pressure may force firms to optimise costs and reallocate internal resources (Barney, 1991). In the longer term, guaranteed procurement volumes can stabilise demand expectations, expand effective market size, and improve cash flow predictability, thereby supporting sustained innovation activities and encouraging firms to adopt collaborative or open innovation strategies (Chesbrough, 2003). The net effect of centralised procurement on innovation is therefore inherently dynamic and potentially heterogeneous across firms.

Existing international experience, such as Group Purchasing Organizations (GPOs) in the United States, highlights both the cost-saving potential and the innovation risks associated with centralised procurement (Huang & Tao, 2020). Unlike GPOs, which operate as third-party coordinators, China’s centralised procurement is directly led by government agencies that simultaneously act as purchasers and payers, creating stronger price-downward pressure and potentially greater profit compression (Long et al., 2022). While a growing body of empirical research finds that China’s centralised procurement policy has, on average, promoted pharmaceutical innovation, the evidence remains mixed regarding short-term versus long-term effects, underlying transmission mechanisms, and firm-level heterogeneity. Most studies focus on early policy stages, pay limited attention to dynamic effects, and insufficiently explore how cost efficiency and operational efficiency mediate innovation responses across different ownership types, firm sizes, and business domains (Chen et al., 2024; Gu & Zhuang, 2023; Li et al., 2023, 2024; Wu, 2023).

This study aims to fill these gaps by systematically examining the impact of the National Drug Centralized Procurement Policy on the innovation capacity of listed pharmaceutical firms in China. Using firm-level panel data and a multi-period difference-in-differences (DID) framework, we analyse both innovation input and innovation output, investigate dynamic policy effects, and identify key transmission channels through cost efficiency and operational efficiency. Furthermore, we explore heterogeneity across ownership structure, firm size, and pharmaceutical subsectors. By doing so, this paper provides new evidence on how volume-based procurement reshapes firms’ innovation incentives and contributes to a more nuanced understanding of the long-term innovation consequences of large-scale health system reforms.

## 2. Methods

### 2.1. Data sources and sample selection

This study examines listed pharmaceutical firms in China using a balanced panel dataset covering the period 2015–2023. Following the industry classification of the Wind Financial Terminal, the study sample includes pharmaceutical companies listed on the Shanghai and Shenzhen A-share markets.

Firm-level information on basic characteristics, operational performance, and financial indicators was obtained from the Wind Financial Terminal (WIND).^1^ Data on invention patents were drawn from the China Research Data Services Platform (CNRDS).^2^ These two data sources are widely used in empirical studies of Chinese firms and provide standardised and reliable information for longitudinal analysis.

To ensure data quality and comparability, several exclusion criteria were applied. Firms designated as ST, *ST, or PT were excluded due to abnormal financial conditions and regulatory risk. Firms with substantial missing data were also removed. To mitigate the influence of outliers, all continuous variables were winsorized at the 5% level.

To isolate the policy’s effect from the noise of financial distress, firms designated as ST, *ST, or PT^3^ are excluded from the primary analysis. However, to address potential concerns regarding sample selection and ensure generalizability of the findings, this study also performed a robustness check using a sample including all ST/*ST/PT firms (see Supplemental Appendix Section 6).

Before screening, the initial sample comprised of an unbalanced panel of 246 pharmaceutical companies listed on the A-share market, covering the period of 9 years (2,214 raw observations). To mitigate the influence of financial distress and regulatory risks, we first excluded 37 firms designated as ST, *ST, or PT. To ensure data integrity, we then excluded 49 firms with missing values for key variables. The final primary analytical sample consists of a balanced panel of 160 firms (1,440 observations).

### 2.2. Innovation measures

This study evaluates the technological innovation capability of pharmaceutical firms using two dimensions: innovation input and innovation output (Lai et al., 2021).

Innovation Input (Y_1_): The extent to which a firm invests in research and development (R&D) is captured by the ratio of total R&D expenditure to operating revenue (Deng et al., 2018; VanderPal, 2015), as reported in audited annual filings And it reflects the firm's commitment to innovation as a percentage of its overall financial resources.

Innovation Output (Y_2_): The number of invention patents granted to a firm in a given year is used to measure its innovation output (Gu & Zhuang, 2023; Li et al., 2023), and it provides a tangible measure of a firm's ability to translate R&D efforts into innovative products or technologies.

Specifically, following Eberhardt et al. (2017), this study focuses on authorised invention patents to ensure that the measure captures substantive innovation. Unlike utility models or design patents, invention patents undergo a rigorous substantive examination for novelty and inventiveness, which minimises the potential distortion caused by strategic or low-quality patenting behaviours.

These two variables, innovation input and innovation output, together form a comprehensive assessment of a pharmaceutical firm's innovation capacity.

### 2.3. Treatment and control groups

The identification strategy relies on variation in firms’ exposure to the NDCPP, which was implemented in a staggered and phased manner. Firms are classified into treatment and control groups based on their participation status in centralised procurement and the timing of policy exposure. It is important to note that the NDCPP’s regulatory constraints apply exclusively to successful bidders. Non-winning firms are not obliged to fulfil the policy’s price or volume requirements.

The treatment group consists of pharmaceutical firms that successfully won bids in centralised procurement projects during the study period. Participation in centralised procurement is firm-specific and occurs at different points in time, reflecting the phased rollout of the policy across pilot rounds (e.g. the ‘4 + 7’ pilot), alliance expansions, and subsequent procurement batches. Once a firm wins a procurement bid, it is considered exposed to the policy from that year onward.

The control group includes pharmaceutical firms that did not win any centralised procurement bids throughout the sample period. These firms were not directly subject to the pricing and volume constraints imposed by the procurement policy and therefore provide a counterfactual for assessing how innovation outcomes would have evolved in the absence of policy exposure.

The use of ‘winning status’ as the primary treatment indicator is justified by the immediate and mandatory nature of the contractual obligations imposed on winning firms. While the NDCPP may exert spillover effects on non-winning firms (the control group), such responses are typically voluntary and strategic. From an econometric perspective, if non-winning firms also increase R&D investment in response to the changing market environment, our staggered DID estimates would represent a lower bound of the policy’s true effect. Furthermore, the inclusion of firm-level fixed effects mitigates concerns regarding systematic differences in competitive tiers between the treatment and control groups by absorbing time-invariant firm-specific characteristics.

To operationalise this design, a time-invariant indicator (Treat_i_) is defined, taking the value of 1 if firm i ever participated in centralised procurement during the sample period, and 0 otherwise. A time-varying indicator (Time_it_) captures the onset of policy exposure, taking the value of 1 in years t greater than or equal to the firm’s first bid-winning year, and 0 otherwise. The interaction of these two indicators forms the staggered DID treatment variable, which equals 1 only for treated firms in their post-treatment periods.

This construction allows firms to enter the treatment group at different times and enables the estimation of the Average Treatment Effect on the Treated (ATT) by comparing changes in innovation outcomes before and after policy exposure between treated and control firms, while controlling for firm fixed effects and year fixed effects.

### 2.4. Staggered Difference-in-Differences (DID) Model

This study adopts a Staggered Difference-in-Differences (DID) model to assess the net impact of the NDCPP on the innovation capabilities of pharmaceutical firms. Given the gradual and phased implementation of the policy (e.g. ‘4 + 7’ pilot, alliance expansion, and subsequent batches), firms were exposed to the policy at different times, creating variation in treatment timing. This staggered treatment would cause significant estimation bias if a traditional two-period DID model were used, which assumes all firms are treated at the same time.

To accurately capture the changes in innovation for firms that entered the policy intervention at different times, this study follows the methodology of Beck (Beck et al., 2010) and Goodman-Bacon (Goodman-Bacon, 2021) by implementing a Staggered DID model for the baseline regression analysis. Staggered difference-in-differences model has been widely applied in health policy research globally, particularly in settings where policies are implemented at different times across regions or units (Freedman et al., 2023). Recent applications include evaluations of Medicaid expansion and healthcare payment reforms, where staggered adoption is a common institutional feature (Thome et al., 2024).

This approach allows firms to enter the treatment at different points in time, and by controlling for both firm fixed effects and year fixed effects, it estimates the Average Treatment Effect on the Treated (ATT).

The specific model is as follows:

$$\mathrm{Innovatio}n_{\mathrm{it}}=\alpha +\mathrm{\beta DI}D_{\mathrm{it}}+\mathrm{\gamma Contro}l_{\mathrm{it}}+\mu_{i}+\lambda_{t}+\varepsilon_{\mathrm{it}}$$

where:

- Innovation_it_: Represents the innovation capacity of firm i in year t, measured by innovation input (Input) and innovation output (Output) respectively.
- DID_it_: The core explanatory variable, a binary indicator. If firm i participated in a procurement project in year t or earlier, DID_it_ = 1; otherwise, DID_it_ = 0. This variable captures the net effect of the centralised procurement policy intervention on the firm’s innovation.
- Control_it_: A set of time-varying control variables that influence firm innovation, such as firm size and leverage.
- μ_i_: Firm fixed effects, which control for time-invariant characteristics of the firms (e.g. firm culture, location). This substitutes for the traditional Treat_i_ variable in a simpler DID model.
- λ_t_: Year fixed effects, which control for macroeconomic shocks or other time-varying factors (e.g. economic cycles, policy changes) that affect all firms equally. This substitutes for the traditional Post_t_ variable.
- ϵ_it_: The error term.

The coefficient β on the DID_it_ term represents the Average Treatment Effect on the Treated (ATT), capturing the policy’s impact on firms that were exposed to the procurement policy. A positive β indicates a beneficial effect on innovation, while a negative β suggests a detrimental effect.

### 2.5. Control variables

Following prior empirical studies on firm innovation and policy evaluation (Chen et al., 2024; Li et al., 2024; Li & Xu, 2024), this study includes a set of firm-level control variables to account for observable characteristics that may influence innovation outcomes.

Specifically, four time-varying control variables are incorporated. Firm size (Size) is measured by the logarithm of total assets and captures scale effects and resource availability for innovation activities. Financial leverage (Lev), defined as the ratio of total liabilities to total assets, reflects firms’ financing constraints and risk exposure, which may affect R&D investment decisions. Government subsidies (Gov) are included to account for public financial support that may directly stimulate innovation or offset cost pressures induced by policy interventions. Ownership concentration (Top), measured by the shareholding ratio of the largest shareholder, captures corporate governance characteristics that may shape firms’ strategic orientation toward long-term innovation.

All control variables vary over time and are included alongside firm fixed effects and year fixed effects in the regression models.

### 2.6. Supplemental analyses

To conserve space, several supplemental analyses are reported in the Supplemental Material. Supplemental Appendix 1 reports robustness checks using a Propensity Score Matching combined with Difference-in-Differences (PSM-DID) approach to address potential sample selection bias. Supplemental Appendix 2 presents mechanism (mediation) analyses, examining whether the impact of the NDCPP on firm innovation operates through cost efficiency and operational efficiency. Supplemental Appendix 3 provides heterogeneity analyses, exploring differential policy effects across firms by ownership structure, firm size, and business domain.

## 3. Results

### 3.1. Descriptive statistics

Table 1 reports the descriptive statistics for all variables used in the analysis, based on our final analytical sample of 160 listed pharmaceutical firms (1,440 observations), including the variables’ means, standard deviations, and minimum and maximum values. On average, firms allocate 5.35% of operating revenue to R&D investment, indicating a moderate level of innovation input across listed pharmaceutical firms, with substantial dispersion reflecting pronounced heterogeneity in R&D intensity within the industry. Innovation output, measured by the number of invention patents granted, has a mean value of 5.9639 and exhibits a markedly right-skewed distribution, suggesting that most firms generate relatively few patents while a small number of highly innovative firms account for a disproportionate share of total patenting activity.

**Table 1.** Descriptive statistics of key variables.

Variables	Observations	Average value	Standard deviation	Min	Max
Input	1,440	0.0535	0.0339	0.0093	0.1362
Output	1,440	5.9639	7.4518	0	26
Cost	1,440	0.4337	0.2087	0.0591	0.7676
InvTrn	1,440	2.8474	1.5153	0.7784	6.6312
Size	1,440	5.6070	0.4232	4.8693	6.3982
Lev	1,440	0.3239	0.1524	0.1036	0.6267
Top	1,440	0.5038	0.1460	0.2513	0.7791
Govsub	1,438	3.2325	0.5482	2.1831	4.1682

Notes: This table reports descriptive statistics for the primary analytical sample of 160 firms (1,440 observations) from 2015 to 2023. Input refers to R&D intensity (R&D expenses divided by operating revenue); Output is the number of authorised invention patents; Cost represents the operating cost ratio; InvTrn is the inventory turnover ratio; Size is the natural logarithm of total assets; Lev is the leverage ratio (total liabilities divided by total assets); Top denotes ownership concentration (shareholding ratio of the largest shareholder); Govsub is the natural logarithm of government subsidies.

With respect to the mediating variables, the average operating cost ratio is 0.4337, indicating notable variation in cost structures across firms. The mean inventory turnover ratio is 2.8474 times per year, with a relatively large standard deviation, reflecting substantial differences in operational efficiency among pharmaceutical firms. Overall, the distributional characteristics of the key variables are consistent with the pharmaceutical industry’s stylised features, characterised by concentrated innovation output alongside pronounced heterogeneity in firms’ operational performance.

### 3.2. Baseline staggered DID regression

Table 2 presents the baseline estimates of the impact of the NDCPP on pharmaceutical firms’ innovation outcomes. The models are estimated using a staggered Difference-in-Differences (DID) specification, controlling for firm fixed effects and year fixed effects, and results are reported both with and without additional time-varying control variables.

**Table 2.** Benchmark regression results.

Variables	Input		Output	
	(1)	(2)	(3)	(4)
DID	0.0118***	0.0118***	0.769	0.847*
	(6.06)	(6.16)	(1.56)	(1.73)
Size		0.0111**		5.971***
		(2.11)		(4.79)
Lev		−0.0125*		−3.285**
		(−1.74)		(−2.54)
Top		0.0064		0.587
		(0.94)		(0.52)
Govsub		0.0013		1.261***
		(0.66)		(2.83)
_cons	0.0519***	−0.0137	5.861***	−30.94***
	(108.82)	(−0.52)	(47.31)	(−4.63)
Firm FE	Yes	Yes	Yes	Yes
Year FE	Yes	Yes	Yes	Yes
R^2^	0.8094	0.8118	0.7435	0.7547
Adj. R^2^	0.7843	0.7862	0.7095	0.7214
N	1440	1438	1440	1438

Notes: The dependent variables are R&D intensity (Input) and the number of authorised invention patents (Output). Columns (1) and (3) report results without control variables, while Columns (2) and (4) include control variables of Size, Lev, Top and Govsub. All models include firm fixed effects and year fixed effects to control for time-invariant firm characteristics and macro-level temporal shocks. Robust standard errors clustered at the firm level are reported in parenthese. *, **, and *** indicate statistical significance at the 10%, 5% and 1% levels, respectively.

The results of Columns (1) and (2) indicate a statistically significant positive effect of the policy on firms’ innovation input. As shown in Column (2), the coefficient for innovation input is 0.0118 (*p* < 0.01), implying that the implementation of the NDCPP led to an abosulte increase of 1.18 percentage points in firms’ R&D expense-to-revenue ratio. To put this in perspective, this represents a 22.06% increase relative to the overall sample mean of 5.35%, indicating a substantial strategic pivot toward innovation. Specifically, compared to the control group, firm selected for the NDCPP demonstrate a stronger propensity to allocated existing resources toward R&D activities. This finding implies that exposure to the centralised procurement policy is associated with a higher proportion of operating revenue allocated to R&D activities.

Consistent with the increase in innovation input, the policy is also associated with improved innovation output. Column (4) shows that the DID coefficient for innovation output is 0.847 (*p* < 0.10), suggesting that on average treated firms obtained 0.847 additional invention patents per year following the policy shock. This corresponds to a 14.2% increase over the sample mean, confirming that the policy not only simulated innovation input but also successfully translated these investments into tangible intellectual property rights.

Taken together, these results suggest that the centralised procurement policy is positively associated with both innovation investment and subsequent innovative output among participating pharmaceutical firms.

### 3.3. Dynamic effects analysis

To examine the dynamic effects of the NDCPP, we extend the baseline staggered DID specification by interacting the treatment indicator with a set of event-time dummies. Specifically, Current denotes the year of policy implementation, while Post 1 to Post 4 denote the first through fourth years following a firm’s initial exposure to the policy. The estimated coefficients therefore capture the evolution of policy effects over time relative to the pre-treatment period. The results are reported in Table 3. The results are reported in Table 3.

**Table 3.** Dynamic effect regression results.

Variables	Input	Output
	(1)	(2)
Current	0.00555**	0.494
	(2.13)	(0.92)
Post 1	0.00889***	1.218*
	(2.96)	(1.91)
Post 2	0.0134***	1.508*
	(4.33)	(1.72)
Post 3	0.0170***	0.711
	(4.18)	(0.79)
Post 4	0.0146***	0.0246
	(2.86)	(0.01)
Firm FE	Yes	Yes
Year FE	Yes	Yes
Control	Yes	Yes
N	1438	1438

Notes: This table presents the results of the event study analysis to test the dynamic impacts of the NDCPP. The variable ‘Current’ represents the year of policy implementation, while ‘Post 1’ through ‘Post 4’ denote the subsequent years following the treatment. Input refers to R&D intensity (R&D expenses divided by operating revenue), and Output refers to the number of authorised invention patents. Control variables (Size, Lev, Top, and Govsub), Firm fixed effects, and year fixed effects are included in all models. Robust standard errors clustered at the firm level are reported in parentheses., *, and *** indicate statistical significance at the 10%, 5%, and 1% levels, respectively.

Column (1) of Table 3 presents the dynamic effects on innovation input. The estimated coefficients reveal a pattern of immediate response and continuous enhancement in R&D investment intensity. Specifically, the coefficient for the implementation year (Current) is 0.00555 (*p* < 0.05), suggesting that pharmaceutical firms respond positively and immediately to the policy shock rather than experiencing a ‘short-term inhibition’. From the first to the third post-treatment years (Post 1 to Post 3), the coefficients exhibit a steady and highly significant upward trend (0.00889, 0.0134, and 0.0170, respectively; all *p* < 0.01). This indicates that the incentive effect of the procurement reform accumulates and amplifies over time. As the ‘price-for-volume’ mechanism stabilises market expectations, firms undergo a rapid strategic transformation, reallocating redundant marketing funds – previously saved through the reduction of sales expenses – into R&D activities to ensure long-term survival and competitiveness. Although the coefficient slightly retreats to 0.0146 in the fourth year (Post 4), it remains significant at the 1% level, indicating a sustained high level of innovation investment.

Column (2) of Table 3 reports the dynamic effects on innovation output. The estimated coefficients are significantly positive in the early post-treatment stages, with significant effects observed in Post 1 (1.218, *p* < 0.10) and Post 2 (1.508, *p* < 0.10). This rapid growth in innovation output likely stems from a ‘stress response’ where firms accelerate the conversion of existing R&D pipeline projects or make marginal improvements to mature technologies to secure patents remain positive, they appear less statistically significant compared to the early stage. This can be attributed to the natural lag in new drug development: the massive ‘policy-driven incremental’ R&D investment observed in Input requires a longer cycle (typically 3–5 years) to translate into new batches of high-quality invention patents.

It should be noted that some firms in the sample were exposed to the policy relatively late (e.g. after 2021), resulting in fewer observations for the fourth and fifth post-treatment years. Consequently, the estimates for Post 4 are primarily driven by firms affected by earlier procurement rounds. This data limitation does not affect the identification of the average treatment effect but should be borne in mind when interpreting the longer-horizon dynamic estimates.

### 3.4. PSM-DID robustness check

Table 4 reports the results of the Propensity Score Matching combined with Difference-in-Differences (PSM-DID) analysis. After matching treated and control firms on observable pre-treatment characteristics, the estimated policy effects remain positive and statistically significant. Specifically, Column (1) shows that the DID coefficient for innovation input is 0.012 and significant at the 1% level, while Column (2) indicates that the DID coefficient for innovation output is 0.852 and significant at the 10% level. These estimates are consistent in both sign and magnitude with the baseline DID results, suggesting that the main findings are not driven by observable selection bias.

**Table 4.** PSM-DID regression results.

Variables	Input	Output
	(1)	(2)
DID	0.012***	0.852*
	(0.002)	(0.497)
Size	0.010*	5.995***
	(0.005)	(1.263)
Lev	−0.011	−3.326**
	(0.007)	(1.299)
Top	0.009	0.405
	(0.007)	(1.182)
Govsub	0.001	1.237***
	(0.002)	(0.449)
_cons	−0.009	−30.953***
	(0.027)	(6.769)
Firm FE	Yes	Yes
Year FE	Yes	Yes
R^2^	0.812	0.747
N	1416	1416

Notes: This table reports the results using the Propensity Score Matching Difference-in-Differences (PSM-DID) method to mitigate potential selection bias between the treatment and control groups. The matching was performed using a nearest-neighbor approach based on firm-level characteristics. The sample size (N = 1,416) is slightly smaller than the baseline sample because observations failing to meet the common support condition were excluded during the matching process. Input is R&D intensity; Output is the number of authorised invention patents; Size is firm scale; Lev is leverage; Top is ownership concentration; Govsub is government subsidies. All models include Firm and Year Fixed Effects. Robust standard errors clustered at the firm level are reported in parentheses. *, **, and *** indicate statistical significance at the 10%, 5%, and 1% levels, respectively.

**Figure 1.**
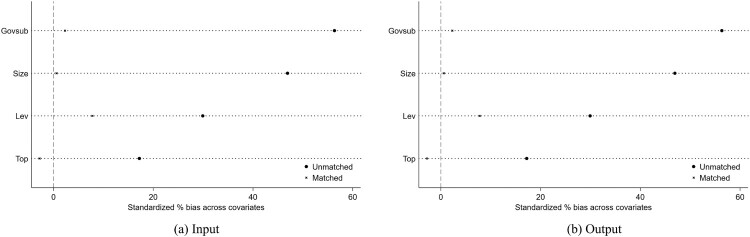
Covariate balance plot. (a) Input. (b) Output.

Figures 1(a and b) present the covariate balance diagnostics before and after matching. Prior to matching, treated and control firms exhibit statistically significant differences across several matching variables. After matching, the standardised differences are substantially reduced and the associated *p*-values increase, indicating no statistically significant differences between the two groups. Overall, the balance tests confirm that the matching procedure effectively improves comparability between treated and control firms, thereby reinforcing the robustness of the baseline estimates.

Supplemental mediation analyses (Supplemental Appendix Tables A1–A2) reveal distinct transmission mechanisms: cost efficiency mediates innovation output, while operational efficiency exerts a complementary mediation effect on innovation input, indicating that firms increased strategic inventory (lowering turnover) to safeguard R&D investment against supply uncertainties. Heterogeneity analyses in Supplemental Appendix Tables A3–A5 show that centralised procurement raises innovation input across firm types, but positive effects on innovation output are observed primarily among private firms, large firms, and Western medicine producers.

To address concerns that the estimated policy effects may be driven by unobserved confounding factors, we conduct placebo tests based on 500 random reassignments of treatment status (Supplemental Appendix Figure A1). The placebo coefficients are centred around zero and largely insignificant, while the baseline estimates lie in the tail of the placebo distributions, supporting the robustness of our main results. We conduct an event-study–based parallel trends test and find no statistically significant differences in innovation input or output between treated and control firms prior to policy implementation, supporting the parallel trends assumption (Supplemental Appendix Figure A1). Post-treatment estimates indicate a sustained positive effect on R&D investment and a short-term positive effect on innovation output.

## 4. Discussion

This study provides a detailed empirical assessment of how China’s NDCPP influences the innovation activities of listed pharmaceutical firms. Using a multi-period difference-in-differences framework, we find evidence suggesting that the policy’s impact on innovation metrics exhibits a clear dynamic pattern characterised by an immediate response and continuous enhancement. By jointly examining innovation input and innovation output, and by explicitly modelling cost efficiency and operational efficiency as mediating channels, the analysis suggests that the policy reshapes firms’ innovation incentives through both price and quantity mechanisms. Moreover, the heterogeneity analyses highlight substantial asymmetries in firms’ innovation responses across ownership structures, firm sizes, and business domains, notably finding that TCM firms have also begun to show significant discernible positive responses in innovation input. Taken together, the findings underscore the complexity of policy-induced innovation effects and indicate that centralised procurement may serve as an immediate catalyst for strategic transformation, triggering proactive organisational responses that stimulate innovation-related efforts from the very onset of policy implementation.

The results of this study both corroborate and extend existing empirical evidence on centralised procurement and pharmaceutical innovation. Consistent with prior studies, we find that the centralised procurement policy is positively associated with higher innovation input and patent output in the aggregate, aligning with the conclusions of previous studies (Chen et al., 2024; Gu & Zhuang, 2023). Moreover, by incorporating a dynamic specification, this study goes beyond the static average effects documented in much of the literature and identifies a notable ‘immediate response – continuous enhancement’ trajectory. Unlike previous findings that suggested a ‘short-term suppression’ (Li et al., 2023; Wang, 2022), our results suggest that firms respond to the ‘price-for-volume’ shock with an immediate ‘innovation-for-survival’ instinct. Our finding of an immediate positive response may aligns with recent empirical evidence (Gu & Zhuang, 2023) reflect a rapid reallocation of resources – shifting redundant marketing funds saved by the policy directly into R&D activities. Over time, as firms adapt to the new procurement environment, the stabilising effects of guaranteed purchase volumes and strategic operational restructuring (characterised by increased inventory turnover) appear to dominate, leading to a sustained engagement with innovation incentives (Ahmad et al., 2025; Wu et al., 2025). Furthermore, this study contributes to the literature by offering a more nuanced mechanism analysis. While earlier researches have emphasised profitability (Wu, 2023), government subsidies (Chen et al., 2024), or financing constraints (Li et al., 2023) as transmission channels, our findings point to cost efficiency and operational efficiency playing distinct and complementary mediating roles. Cost optimisation appears to enhance firms’ ability to convert existing R&D efforts into measurable innovation outputs, whereas strategic adjustments in operational efficiency – specifically, sacrificing short-term inventory turnover speed to ensure supply stability – support sustained R&D investment by securing the operational foundation against supply risks. This dual-path mechanism is consistent with evolutionary economics (Nelson & Winter, 1982) and enriches the empirical understanding of how institutional pressure can translate into observable innovation outcomes.

The findings of this study yield several important policy implications. First, the results suggest that policymakers consider that the innovation effects of centralised procurement are highly responsive and should focus on consolidating the immediate momentum generated by the policy. The fact that R&D investment surges upon policy implementation suggests that the ‘substitution effect’ between marketing and R&D could potentially serve as a tool for industry upgrading (Liu & Fang, 2024). Second, to strengthen the sustained innovation-enhancing effects, complementary policies should be implemented to ensure the sustainability and especially the quality of the observed innovation surge. Targeted R&D subsidies or tax incentives should be considered to serve as risk-sharing mechanisms. These instruments can mitigate the heightened financial vulnerability of firms operating under thin margins and guide them towards high-quality and sustainable innovation. Third, the pronounced heterogeneity suggests that procurement design could be further refined. Importantly, the discovery of a significant positive response in innovation input among TCM firms suggests the policy’s ‘push effect’ may extend beyond chemical pharmaceuticals. Finally, acknowledging that centralised procurement appears to favour efficiency-driven and incremental innovation, additional institutional arrangements may be needed to encourage breakthrough innovation, such as strengthening intellectual property protection and improving market access pathways for innovative drugs.

Despite its contributions, this study is subject to three limitations that warrant caution in interpreting the results. First, the analysis is based on A-share listed pharmaceutical manufacturing firms, which may not fully represent the behaviour of small and unlisted firms, which often face more severe financing constraints and may respond differently to procurement policies. Both firms unparticipated in the NDCPP and lost during the bidding process are grouped in untreated in this study because of unavailable bidding information. Future researches could be conducted based on information disclosure with more granular, batch-specific bidding data. Second, while R&D intensity and authrized patent counts provide measurable indicators of innovation efforts, they primarily capture innovation quantity rather than long-term innovation quality. The absence of high-value metrics – such as breakthrough patent citations, clinical pipeline progression, or New Drug Approvals (NDA) – may understate the heterogeneity in the substantive quality of innovation. Third, the research window (2018–2023) is relatively short compared to the typically decadal cycle of pharmaceutical R&D. Future research should adopt a more longitudinal perspective, tracking clinical-stage milestones and NDAs as more data become available to provide a more comprehensive assessment of innovation depth.

In conclusion, this study demonstrates that China’s NDCPP exerts a complex and dynamic influence on corporate strategy. While the policy initially imposes price-driven pressures, it triggers a strategic reorientation driven by resource reallocation and a substitution effect from marketing to R&D. These immediate responses, coupled with long-term effects mediated through stabilised demand and cost optimisation, ultimately promote innovation input and substantive technical output. The evidence supports the view that centralised procurement can serve as a catalyst for innovation-led trajectory across the entire sector, including traditional Chinese medicine. While our findings capture a significant shift in resource allocation and intermediate technical milestones (authorised invention patents), we acknowledge that a full transition to an innovation-driven model is a long-term evolution that will ultimately be validated by future clinical-stage breakthroughs. By revealing the dual price–quantity mechanisms, heterogeneous firm responses, and efficiency-based transmission channels, this study contributes to the literature on innovation economics and health policy and offers actionable insights for designing procurement policies that balance cost control with the long-term goal of fostering high-quality pharmaceutical innovation.

## Supplementary Material

Supplemental Material

## Data Availability

The data used in this study are derived from commercial databases and publicly available sources. Due to licensing restrictions, the datasets are not publicly available but are available from the corresponding author upon reasonable request.

## Footnotes

1 Wind Financial Terminal is a comprehensive database providing audited financial statements and corportate governanace information for all A-share listed firms in China. Wind is one of the most authoritative provider of financial data for China’s capital market, equivalent to Bloomberg or Refinitiv.

2 CNRDS is a specialized academic platform widely utilized in economic and management research for its high-quality, verified data on intellectual property rights in China.

3 Under the listing rules of the Chinese A-share market, the labels ST (Special Treatment), *ST (Delisting Risk), and PT (Particular Transfer) are applied to firms experiencing serious financial distress or potential delisting risk due to consecutive losses or other abnormal conditions.
