# Supplementary material for: The impact of China’s National Drug Centralized Procurement Policy on pharmaceutical firm innovation: evidence from a staggered difference-in-differences analysis
Source: J Pharm Policy Pract. 2026 Jun 8;19(1):2680235. doi: 10.1080/20523211.2026.2680235 (PMC13248488; doi:10.1080/20523211.2026.2680235)
Supplement: Supplemental Material [file JPPP_A_2680235_SM6788.zip › Supplemental material/Revised_supplementary_material_clean_version.docx]

**Appendix**

# 1. PSM-DID method

To address potential sample selection bias and refine the estimation of treatment effects, this study employs the Propensity Score Matching (PSM) combined with Difference-in-Differences (DID) (PSM-DID) methodology as a robustness check. The PSM approach (Rosenbaum & Rubin, 1983) helps mitigate systematic differences between the treatment and control groups that might exist based on their pre-treatment characteristics.

## 1.1. Propensity score matching procedure

In this study, firm size, capital structure, and other firm-specific characteristics that are unlikely to be immediately affected by the policy intervention are selected as covariates for estimating the propensity score. A Logit model is used to estimate the probability of each firm being exposed to the procurement policy.

After estimating the propensity scores, firms in the treatment and control groups are matched based on their scores using methods such as nearest-neighbor matching and caliper matching (with a caliper value of 0.05). Firms that do not match well are excluded from the analysis. Following this, the DID analysis is re-executed on the matched sample to test the robustness of the results.

## 1.2 Matching procedure

The 1:1 nearest-neighbor matching method, combined with radius matching (caliper = 0.05), is applied to ensure that the matched treatment and control groups are comparable. Matching is done within the common support region, and without replacement to enhance the quality and robustness of the matched sample.

# 2. Mediation effect test models

The impact of the NDCPP on firm innovation operates through both price and volume mechanisms. In the short term, substantial price reductions may compress profit margins and create downward pressure on R&D investment. In the longer term, however, guaranteed procurement volumes may stabilize production expectations and allow firms to adjust internal resource allocation. In this study, these dynamic effects are hypothesized to operate through two mediating channels: cost efficiency and operational efficiency.

First, centralized procurement may promote cost optimization, freeing internal resources and thereby supporting innovation activities. To capture this mechanism, cost efficiency (Cost) is measured using the operating cost ratio, defined as operating costs relative to operating revenue.

Second, centralized procurement may improve inventory management and liquidity by stabilizing demand and accelerating inventory turnover, which in turn may support sustained R&D investment. Operational efficiency (InvTrn) is therefore measured using the inventory turnover ratio.

These two mediating variables are incorporated into stepwise regression models to examine whether and how centralized procurement affects firm innovation through intermediate efficiency improvements.

Drawing on the classic three-step mediation testing procedure proposed by Wen and Ye (2024) and the causal steps approach of Baron and Kenny (1986), this study examines the mediation effect of cost efficiency and operational efficiency in the relationship between the National Drug Centralized Procurement Policy and innovation input and output among the treated pharmaceutical firms. Cost efficiency is measured using the operating cost ratio, while operational efficiency is measured using the inventory turnover ratio.

## 2.1 Cost efficiency as a mediator

To examine the mediation effect of cost efficiency, the following regression models are used:

**Cost Efficiency Model:**

$${Cost}_{it}=\alpha_{1}+\beta_{1}{DID}_{it}+\gamma_{1}{Control}_{it}+\mu_{i}+\lambda_{t}+\varepsilon_{it}$$

**Innovation Input Model:**

$${Input}_{it}=\alpha_{2-1}+\beta_{2-1}{DID}_{it}+\beta_{3-1}{Cost}_{it}+\gamma_{2-1}{Control}_{it}+\mu_{i}+\lambda_{t}+\varepsilon_{it}$$

**Innovation Output Model:**

$${Output}_{it}=\alpha_{2-2}+\beta_{2-2}{DID}_{it}+\beta_{3-2}{Cost}_{it}+\gamma_{2-2}{Control}_{it}+\mu_{i}+\lambda_{t}+\varepsilon_{it}$$

- **Cost efficiency model**: Regresses the operating cost ratio (Cost) on the policy variable (DID) and controls;
- **Innovation input model:** Regresses innovation input on the policy, the operating cost ratio, and controls;
- **Innovation output model**: Regresses innovation output on the policy, the operating cost ratio, and controls;

where **β_1_** represents the effect of the policy intervention on the respective mediating variable (cost efficiency), while **β_3-1_** and **β_3-2_** capture the direct effects of the mediators on innovation input and output.

The results from Column (1) of Table A1 show that the DID coefficient is −0.0471 and statistically significant at the 5% level, indicating that exposure to the centralized procurement policy is associated with a reduction in firms’ operating cost ratios, consistent with improved cost efficiency. Column (2) examines innovation input and shows that the coefficient on cost efficiency is negative but statistically insignificant, suggesting that changes in operating costs do not play a mediating role in the policy’s effect on R&D investment intensity. This implies that the observed increase in innovation input operates through channels other than cost efficiency.

**Appendix Table A1**. Cost efficiency mediation stepwise regression results

| Variables | Cost | Input | Output |
| --- | --- | --- | --- |
|  | (1) | (2) | (3) |
| DID | -0.0471** | 0.0117*** | 0.808* |
|  | (-2.32) | (6.12) | (1.65) |
| Cost |  | -0.00221 | -1.963** |
|  |  | (-0.65) | (-2.25) |
| Size | -0.0483** | 0.0111* | 6.015*** |
|  | (-2.37) | (2.12) | (4.83) |
| Lev | -0.0471 | -0.0121* | -2.922** |
|  | (-1.18) | (-1.68) | (-2.24) |
| Top | -0.00567 | 0.0064 | 0.555 |
|  | (-0.13) | (0.93) | (0.49) |
| Govsub | 0.0268 | 0.00135 | 1.260*** |
|  | (1.64) | (0.66) | (2.83) |
| _cons | 0.642*** | -0.0132 | -30.43*** |
|  | (7.67) | (-0.50) | (-4.57) |
| Firm FE | Yes | Yes | Yes |
| Year FE | Yes | Yes | Yes |
| R^2^ | 0.1102 | 0.8119 | 0.7559 |
| adj. R^2^ | 0.0841 | 0.7862 | 0.7225 |
| N | 1438 | 1438 | 1438 |

Notes: This table reports the results of the mediation effect analysis using a stepwise regression approach to examine whether cost efficiency mediates the relationship between the NDCPP and firm innovation. Column (1) examines the impact of the policy shock (DID) on the mediator, Cost (operating cost ratio). Columns (2) and (3) examine the impact on innovation input (Input) and output (Output) while controlling for the mediator. All specifications include firm-level controls (Size, Lev, Top, and Govsub), firm fixed effects, and year fixed effects. Robust standard errors clustered at the firm level are reported in parentheses. *, **, and *** indicate statistical significance at the 10%, 5%, and 1% levels, respectively.

In contrast, Column (3) reveals a statistically significant negative relationship between cost efficiency and innovation output, with a coefficient of −1.963 (p < 0.05), indicating that lower operating cost ratios are associated with higher levels of patenting activity. Combined with the significant policy effect on cost efficiency, these results suggest the presence of a partial mediation effect for innovation output, highlighting cost optimization as an important transmission channel through which centralized procurement enhances firms’ innovative performance. Overall, the findings indicate that cost efficiency primarily affects innovation output rather than innovation input.

## 2.2 Operational efficiency as a mediator

Similarly, the mediation effect of operational efficiency is examined using the following models:

**Operational Efficiency Model:**

$${InvTrn}_{it}=\alpha_{1}+\beta_{1}{DID}_{it}+\gamma_{1}{Control}_{it}+\mu_{i}+\lambda_{t}+\varepsilon_{it}$$

**Innovation Input Model:**

$${Input}_{it}=\alpha_{2-1}+\beta_{2-1}{DID}_{it}+\beta_{3-1}{InvTrn}_{it}+\gamma_{2-1}{Control}_{it}+\mu_{i}+\lambda_{t}+\varepsilon_{it}$$

**Innovation Output Model:**

$${Output}_{it}=\alpha_{2-2}+\beta_{2-2}{DID}_{it}+\beta_{3-2}{InvTrn}_{it}+\gamma_{2-2}{Control}_{it}+\mu_{i}+\lambda_{t}+\varepsilon_{it}$$

- **Operational efficiency model**: Regresses the inventory turnover ratio (InvTrn) on the policy variable (DID) and controls.
- **Innovation input model**: Regresses innovation input on the policy, the inventory turnover ratio, and controls.
- **Innovation output model**: Regresses innovation output on the policy, the inventory turnover ratio, and controls.

where **β_1_** represents the effect of the policy intervention on the respective mediating variable (operational efficiency), while **β_3-1_** and **β_3-2_** capture the direct effects of the mediators on innovation input and output.

Column (1) of Table A2 shows that the DID coefficient is −0.173 and statistically significant at the 5% level, indicating that policy exposure is associated with a significant change in firms’ inventory turnover. Column (2) further demonstrates a statistically significant relationship between operational efficiency and innovation input: the coefficient on inventory turnover is −0.00574 and significant at the 1% level, suggesting that changes in inventory management are associated with adjustments in R&D investment intensity. The negative value suggests that, the inventory turnover ratio of winning enterprises actually declined following the implementation of the procurement policy. This indicates a slower inventory turnover speed and a relative increase in inventory holding level. Meanwhile, as the impact coefficient of inventary turnover on innovation input is also negative, the calculated indirect effect is positive ($\beta_{1}\times\beta_{3-1}>0$). According to the mediation classification procedure proposed by Zhao et al. (2010), since the sign of this indirect effect is consistent with the sign of the direct effect of the procurement policy on innovation input, it demonstrates that the inventory turnover ratio plays a role of Complementary Mediation in this process.

In contrast, Column (3) shows that the coefficient on inventory turnover is positive but statistically insignificant when innovation output is used as the dependent variable. This suggests that operational efficiency does not constitute a significant transmission channel through which centralized procurement affects firms’ patenting outcomes.

**Appendix Table A2.** Operational efficiency mediation stepwise regression results

| Variables | InvTrn | Input | Output |
| --- | --- | --- | --- |
|  | (1) | (2) | (3) |
| DID | -0.173** | 0.0108*** | 0.847* |
|  | (-2.39) | (5.92) | (1.73) |
| InvTrn |  | -0.00574*** | 0.0013 |
|  |  | (-9.09) | (0.01) |
| Size | -0.273 | 0.00950* | 5.971*** |
|  | (-1.18) | (1.87) | (4.76) |
| Lev | 0.840*** | -0.00765 | -3.286** |
|  | (2.82) | (-1.14) | (-2.54) |
| Top | -0.364 | 0.00435 | 0.587 |
|  | (-1.07) | (0.68) | (0.52) |
| Gov | -0.0966 | 0.000795 | 1.261*** |
|  | (-0.97) | (0.40) | (2.82) |
| _cons | 4.623*** | 0.0128 | -30.94*** |
|  | (3.70) | (0.49) | (-4.52) |
| Firm FE | Yes | Yes | Yes |
| Year FE | Yes | Yes | Yes |
| R^2^ | 0.8223 | 0.8235 | 0.7547 |
| adj. R^2^ | 0.7981 | 0.7993 | 0.7212 |
| N | 1438 | 1438 | 1438 |

Notes: This table reports the results of the mediation effect analysis using a stepwise regression approach to examine whether operational efficiency mediates the relationship between the NDCPP and firm innovation. Column (1) examines the impact of the policy shock (DID) on the mediator, InvTrn (inventory turnover ratio). Column (2) and (3) examine the impact on innovation input (Input) and output (Output) while controlling for the mediator. All specifications include firm-level controls (Size, Lev, Top, and Govsub), firm fixed effects, and year fixed effects. Robust standard errors clustered at the firm level are reported in parentheses. *, **, and *** indicate statistical significance at the 10%, 5% and 1% levels, respectively..

# 3. Heterogeneity analysis

To further examine heterogeneous policy effects across different types of firms, this study constructs grouping variables based on ownership structure, firm size, and business domain, which are used in subgroup regression analyses.

## 3.1 Ownership type (SOE)

Based on firm disclosures and the classification provided by the Wind Financial Terminal, firms classified as central or local state-owned enterprises are defined as state-owned enterprises (SOE = 1), while firms classified as private enterprises are defined as non-SOEs (SOE = 0). This variable is used to assess whether differences in governance structures and competitive pressures moderate the policy’s impact on innovation.

Appendix Table A3 indicates that the centralized procurement policy has a statistically significant and positive effect on innovation input for both SOEs and private firms, suggesting that increased R&D investment following policy exposure is a common response across ownership types. However, notable differences emerge with respect to innovation output. While the policy is associated with a significant increase in the number of invention patents among private firms, the corresponding effect for SOEs is statistically insignificant. This finding suggests that, although both ownership types expand R&D investment in response to the policy, private firms are more effective in translating increased innovation input into measurable innovative output, potentially reflecting differences in incentive structures, governance mechanisms, and innovation efficiency.

## 3.2 Firm size category (Scope)

Following the national Bureau of Statistics’ Classification of Large, Medium, Small and Micro Enterprises (2017), combined with WIND classifications, firms categorized as large enterprises are defined as Scope = 1, while firms categorized as medium or small enterprises are defined as Scope = 0. This variable captures heterogeneity related to resource endowments, financing capacity, and the maturity of innovation systems.

Appendix Table A4 shows that the NDCPP has a statistically significant and positive effect on R&D investment intensity for both large and medium-sized pharmaceutical firms, indicating that firms across different size categories increase innovation input after policy exposure. However, differences emerge with respect to innovation output.

**Appendix Table A3**. Regression results of the heterogeneity of the ownership type

| Variables | Input | | Output | |
| --- | --- | --- | --- | --- |
|  | State-owned | Non-state-owned | State-owned | Non-state-owned |
| DID | 0.0104*** | 0.0136*** | 1.062 | 1.844*** |
|  | (3.00) | (5.11) | (1.24) | (2.79) |
| Size | 0.00725 | 0.00835 | 0.357 | 4.929*** |
|  | (0.46) | (1.25) | (0.12) | (3.51) |
| Lev | -0.0251* | -0.00988 | -1.032 | -2.785* |
|  | (-1.93) | (-1.04) | (-0.41) | (-1.72) |
| Top | -0.00976 | 0.00488 | -12.49* | 0.525 |
|  | (-0.40) | (0.64) | (-1.90) | (0.46) |
| Govsub | -0.000761 | 0.00235 | 1.440 | 1.887*** |
|  | (-0.24) | (0.76) | (1.80) | (3.18) |
| _cons | 0.0135 | 0.00778 | 7.324 | -27.21*** |
|  | (0.16) | (0.24) | (0.45) | (-3.60) |
| Firm FE | Yes | Yes | Yes | Yes |
| Year FE | Yes | Yes | Yes | Yes |
| R^2^ | 0.7220 | 0.8035 | 0.8208 | 0.7512 |
| adj. R^2^ | 0.6748 | 0.7756 | 0.7903 | 0.7158 |
| N | 359 | 908 | 359 | 908 |

Notes: This table presents the heterogeneity analysis based on firm ownership. The sample is divided into State-owned and Non-state-owned groups according to the ultimate controller of the firm. The dependent variables are R&D intensity (Input) and the number of authorized invention patents (Output). All models include firm-level controls, firm fixed effects, and year fixed effects. Robust standard errors clustered at the firm level are reported in parentheses. *, **, and *** indicate statistical significance at the 10%, 5%, and 1% levels, respectively.

The policy is associated with a significant increase in patent output among large firms, whereas the corresponding effect for medium-sized firms is statistically insignificant. This pattern suggests that larger firms may possess stronger resource endowments, more mature R&D systems, and greater capacity to convert increased R&D investment into tangible innovation outcomes under centralized procurement pressures.

**Appendix Table A4.** Regression results of the heterogeneity of the firm size

| Variables | Input | | Output | |
| --- | --- | --- | --- | --- |
|  | Medium size | Large size | Medium size | Large size |
| DID | 0.0175** | 0.00998*** | 0.0403 | 0.977* |
|  | (2.36) | (5.10) | (0.06) | (1.75) |
| Size | 0.00195 | 0.0101* | 1.315 | 6.577*** |
|  | (0.12) | (1.84) | (0.81) | (4.64) |
| Lev | 0.00125 | -0.0139* | -1.591 | -3.954** |
|  | (0.07) | (-1.91) | (-0.81) | (-2.47) |
| Top | 0.00271 | 0.0146* | 3.492** | 0.161 |
|  | (0.19) | (1.87) | (2.45) | (0.11) |
| Gov | 0.00289 | 0.000974 | 0.285 | 1.691*** |
|  | (0.57) | (0.44) | (0.60) | (2.92) |
| _cons | 0.0297 | -0.0110 | -7.085 | -35.13*** |
|  | (0.38) | (-0.40) | (-0.85) | (-4.50) |
| Firm FE | Yes | Yes | Yes | Yes |
| Year FE | Yes | Yes | Yes | Yes |
| R^2^ | 0.8149 | 0.8148 | 0.5131 | 0.7452 |
| adj. R^2^ | 0.7798 | 0.7892 | 0.4208 | 0.7100 |
| N | 252 | 1186 | 252 | 1186 |

Notes: This table presents the heterogeneity analysis based on firm size. The sample is divided into Medium-size and Large-size groups according to the Classification of Large, Medium, Small and Micro-sized Enterprises for Statistical Purposes (2017) issued by the National Bureau of Statistics (NBS) of China. The dependent variables are R&D intensity (Input) and the number of authorized invention patents (Output). All models include firm-level controls, firm fixed effects, and year fixed effects. Robust standard errors clustered at the firm level are reported in parentheses. *, **, and *** indicate statistical significance at the 10%, 5%, and 1% levels, respectively.

## 3.3 Business domain (Industry)

Based on WIND industry classifications and firms’ main business activities, firms classified as traditional Chinese medicine producers are defined as Industry = 1, while firms classified as chemical or biological pharmaceutical producers are defined as Industry = 0. This variable is used to identify heterogeneous innovation responses arising from differences in technological pathways, R&D models, and policy adaptability.

**Appendix Table A5**. Regression results of the heterogeneity of the business domain

| Variables | Input | | Output | |
| --- | --- | --- | --- | --- |
|  | Chinese medicine | Chemical/biological pharmaceutical | Chinese medicine | Chemical/biological pharmaceutical |
| DID | 0.00766** | 0.0107*** | 0.378 | 1.089* |
|  | (2.57) | (4.45) | (0.42) | (1.88) |
| Size | 0.00677 | 0.00757 | -1.503 | 7.650*** |
|  | (0.95) | (1.19) | (-0.75) | (5.28) |
| Lev | -0.00807 | -0.0164* | -1.844 | -3.303** |
|  | (-0.99) | (-1.72) | (-0.83) | (-2.10) |
| Top | 0.00140 | 0.00769 | 0.901 | 0.323 |
|  | (0.17) | (0.91) | (0.33) | (0.26) |
| Gov | 0.00147 | 0.00153 | 0.536 | 1.773*** |
|  | (0.64) | (0.52) | (0.72) | (3.08) |
| _cons | -0.00302 | 0.0147 | 12.72 | -41.74*** |
|  | (-0.08) | (0.46) | (1.22) | (-5.35) |
| Firm FE | Yes | Yes | Yes | Yes |
| Year FE | Yes | Yes | Yes | Yes |
| R^2^ | 0.8358 | 0.7798 | 0.7674 | 0.7542 |
| adj. R^2^ | 0.8104 | 0.7484 | 0.7314 | 0.7192 |
| N | 531 | 907 | 531 | 907 |

Notes: This table presents the heterogeneity analysis based on the firms’ business domain. The sample is divided into Western medicine and TCM groups based on Wind Industry Classification Standard (WICS). The dependent variables are R&D intensity (Input) and the number of authorized invention patents (Output). All models include firm-level controls, firm fixed effects, and year fixed effects. Robust standard errors clustered at the firm level are reported in parentheses. *, **, and *** indicate statistical significance at the 10%, 5%, and 1% levels, respectively.

Appendix Table A5 indicates that the NDCPP has a statistically significant and positive effect on both innovation input and innovation output among Western medicine firms. For traditional Chinese medicine (TCM) firms, the results reveal a statistically significant and positive effect on innovation input, although the impact on innovation output remains non-significant. This divergence suggests that firms operating in different technological and innovation regimes respond differently to centralized procurement. Western medicine firms, which typically rely more heavily on standardized R&D processes and patent-driven innovation, appear better positioned to adjust innovation strategies and translate policy-induced pressures into measurable innovation outcomes. By contrast, the significant increase in R&D investment among TCM firms reflects a proactive strategic shift as these firms intensify their efforts in innovation to cope with normalized procurement pressure. However, the lack of a significant increase in patent output may stem from the unique complexities and higher hurdles of the TCM patenting process, as well as the industry’s reliance on incremental innovation pathways that are not always captured by invention patent counts (Zhou et al., 2023).

# 4. Placebo (falsification) test

To assess whether the estimated effect of the National Drug Centralized Procurement Policy on firm innovation is driven by unobserved confounding factors, we conduct a placebo (falsification) test following the approach of Lv et al. (2019). Specifically, we repeatedly reassign treatment status by randomly selecting firms into a pseudo-treatment group and re-estimating the baseline staggered DID model. This procedure is repeated 500 times to generate the empirical distribution of placebo estimates.

Appendix Figure A1(a) and Appendix Figure A1(b) present the distributions of the estimated placebo coefficients for innovation input and innovation output, respectively. The placebo estimates are centered around zero and display an approximately normal distribution, with the vast majority of coefficients statistically insignificant. In contrast, the coefficient estimates from the baseline regressions lie in the right tail of the placebo distributions and represent low-probability outcomes under random assignment.


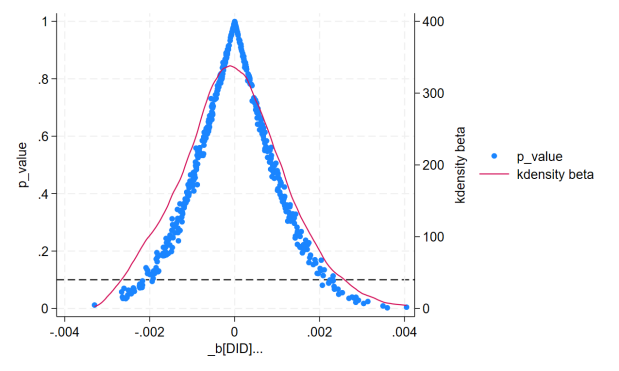

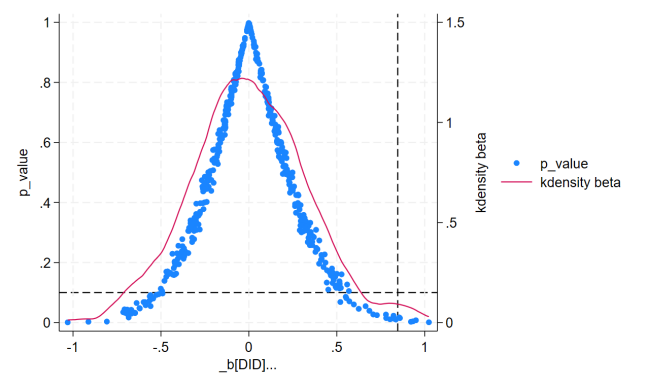


(a) Input (b) Output

**Appendix Figure A1**. Placebo test results

These results suggest that the baseline estimates are unlikely to be driven by unobserved firm-level or time-varying factors unrelated to the procurement policy. Overall, the placebo tests provide additional support for the robustness of the main findings.

# 5. Parallel trends test

To verify the validity of the Difference-in-Differences identification strategy and to mitigate potential bias arising from differential pre-treatment trends, we conduct a parallel trends test comparing innovation outcomes between treated and control firms prior to policy implementation. Following the approach of Beck et al. (2010), we estimate an event-study specification that examines whether treated (winning) and control (non-winning) firms exhibited similar trends in innovation input and innovation output before exposure to the National Drug Centralized Procurement Policy. The estimated coefficients and their 90% confidence intervals are presented in Appendix Figure A2(a) and Appendix Figure A2(b).

As shown in Appendix Figures A2(a) and A2(b), the estimated coefficients for all pre-treatment periods are statistically indistinguishable from zero, with confidence intervals crossing the zero line. This indicates that there were no systematic differences in innovation trends between the treatment and control groups prior to policy implementation. In the post-treatment periods, the confidence intervals for innovation input do not intersect with zero, suggesting a positive policy effect on R&D investment. For innovation output, the coefficients are statistically significant in the early post-treatment periods (post_1 and post_2) but become insignificant in later periods (post_3 and post_4), indicating that the policy’s effect on patenting activity is most pronounced in the early stages following implementation.

Overall, these results support the parallel trends assumption underlying the staggered DID framework and provide further confidence in the causal interpretation of the baseline estimates.


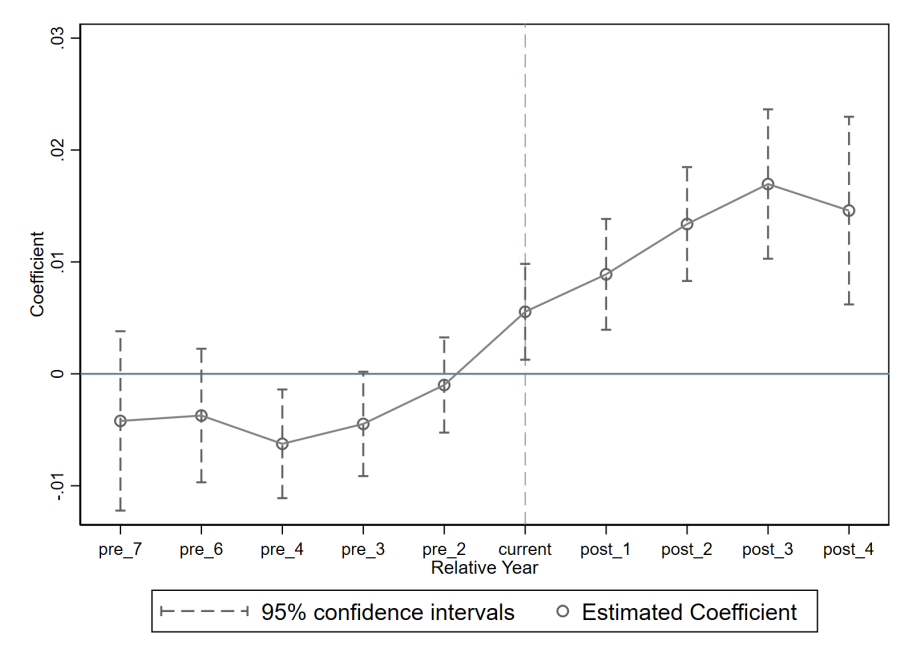

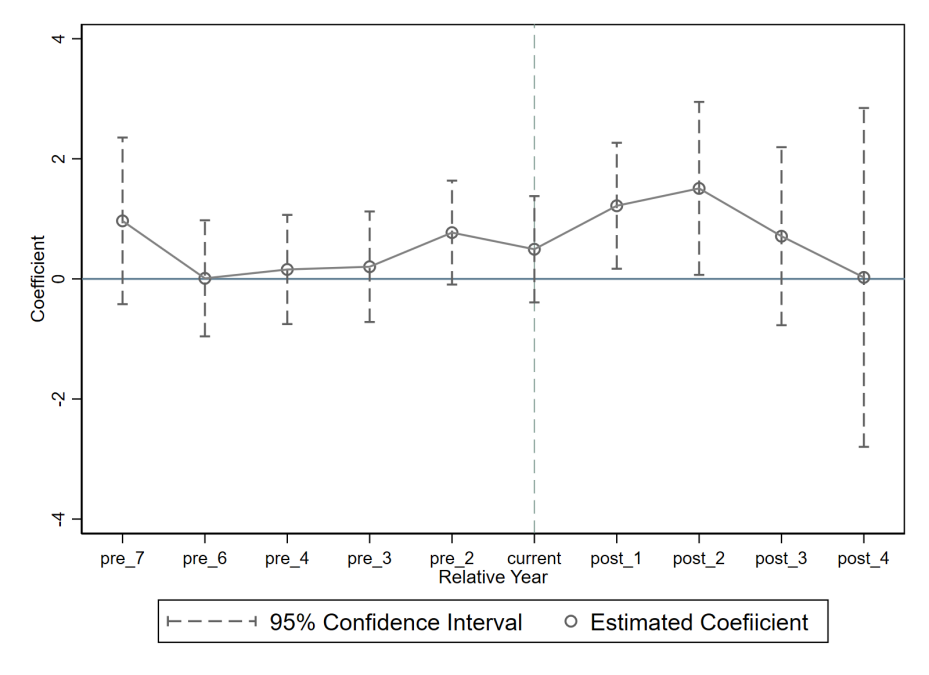


(a) Input (b) Output

**Appendix Figure A2**. Parallel trend test results

# 6. Test for sample selection bias

One potential concern is that the exclusion of financially distressed firms might bias the results. To address this, we extended our analysis to a sample of 197 firms, which includes all listed pharmaceutical companies except those with missing key data. The results, presented in Appendix Table A6, show that the DID coefficients for innovation input (0.0120, p<0.01) and output (0.967, p<0.05) remain positive and significant. This consistency indicates that the pro-innovation impact of the NDCPP is robust across the entire industry, regardless of firms’ financial health status.

**Appendix Table A6.** Benchmark regression results of extended sample

| Variables | Input | Output |
| --- | --- | --- |
|  | (1) | (2) |
| DID | 0.0120*** | 0.9670** |
|  | (5.88) | (2.21) |
| Size | 0.0027 | 5.1410*** |
|  | (0.56) | (5.74) |
| Lev | -0.0049 | -1.963** |
|  | (-0.76) | (-2.09) |
| Top | 0.0133*** | 0.617 |
|  | (3.26) | (1.38) |
| Govsub | 0.0000 | 0.0001 |
|  | (0.69) | (1.78) |
| _cons | 0.0340 | -23.41*** |
|  | (1.32) | (-4.83) |
| Firm FE | Yes | Yes |
| Year FE | Yes | Yes |
| R^2^ | 0.7970 | 0.7526 |
| Adj. R^2^ | 0.7699 | 0.7195 |
| N | 1773 | 1773 |

Notes: This table reports the benchmark regression results using the extended full sample (197 firms, 1,773 observations), which includes firms with abnormal financial conditions (ST, ST, or PT). The dependent variables are R&D intensity (Input) and the number of authorized invention patents (Output). Control variables, firm fixed effects, and year fixed effects are included as in the baseline specification. Robust standard errors clustered at the firm level are reported in parentheses. *, **, and *** indicate statistical significance at the 10%, 5%, and 1% levels, respectively.
