# Supplementary figures and images for: The impact of China’s National Drug Centralized Procurement Policy on pharmaceutical firm innovation: evidence from a staggered difference-in-differences analysis
Source: J Pharm Policy Pract. 2026 Jun 8;19(1):2680235. doi: 10.1080/20523211.2026.2680235 (PMC13248488; doi:10.1080/20523211.2026.2680235)

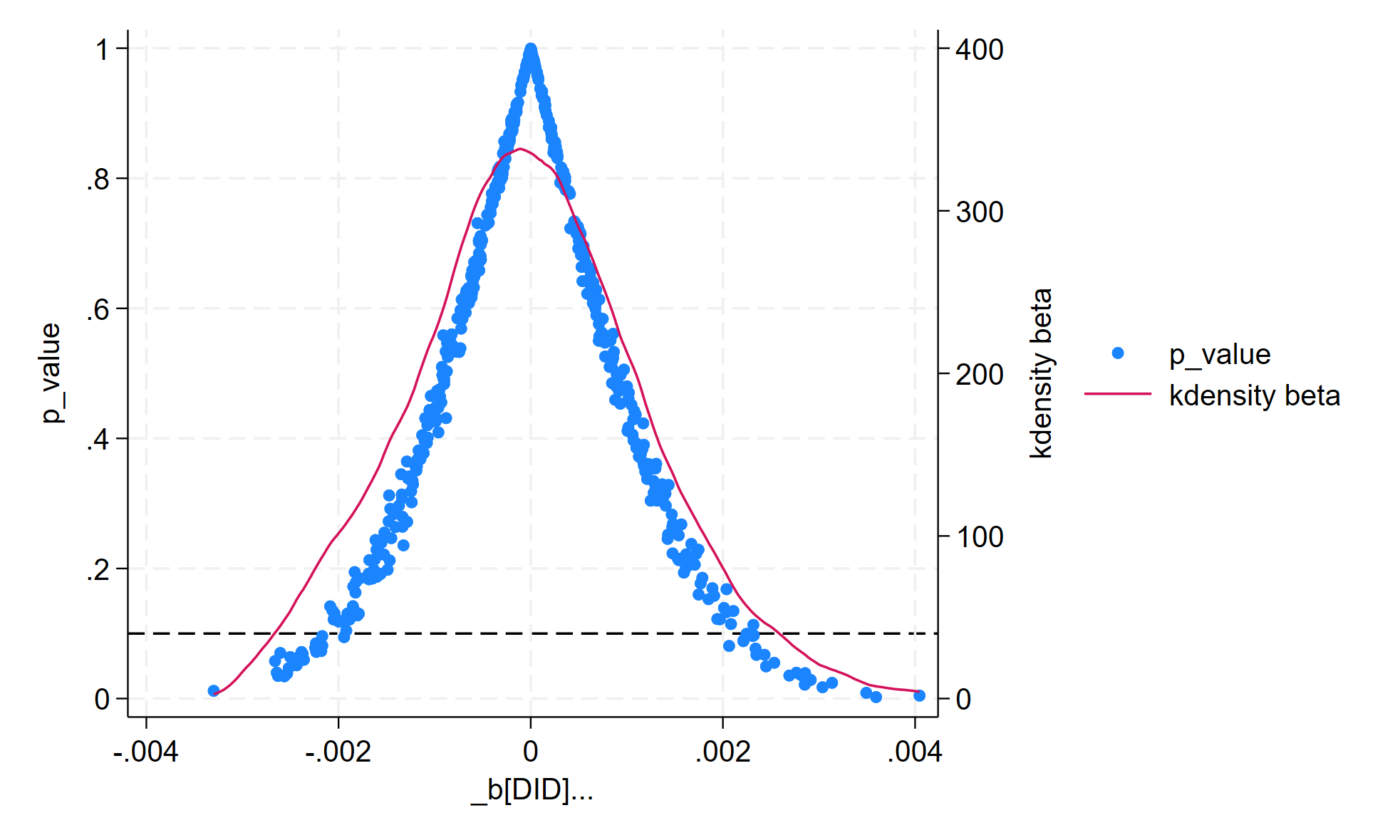

Supplement: Supplemental Material [file JPPP_A_2680235_SM6788.zip › Supplemental material/Appendix_Figure_A1._Placebo_test_results_a_Input.tif]

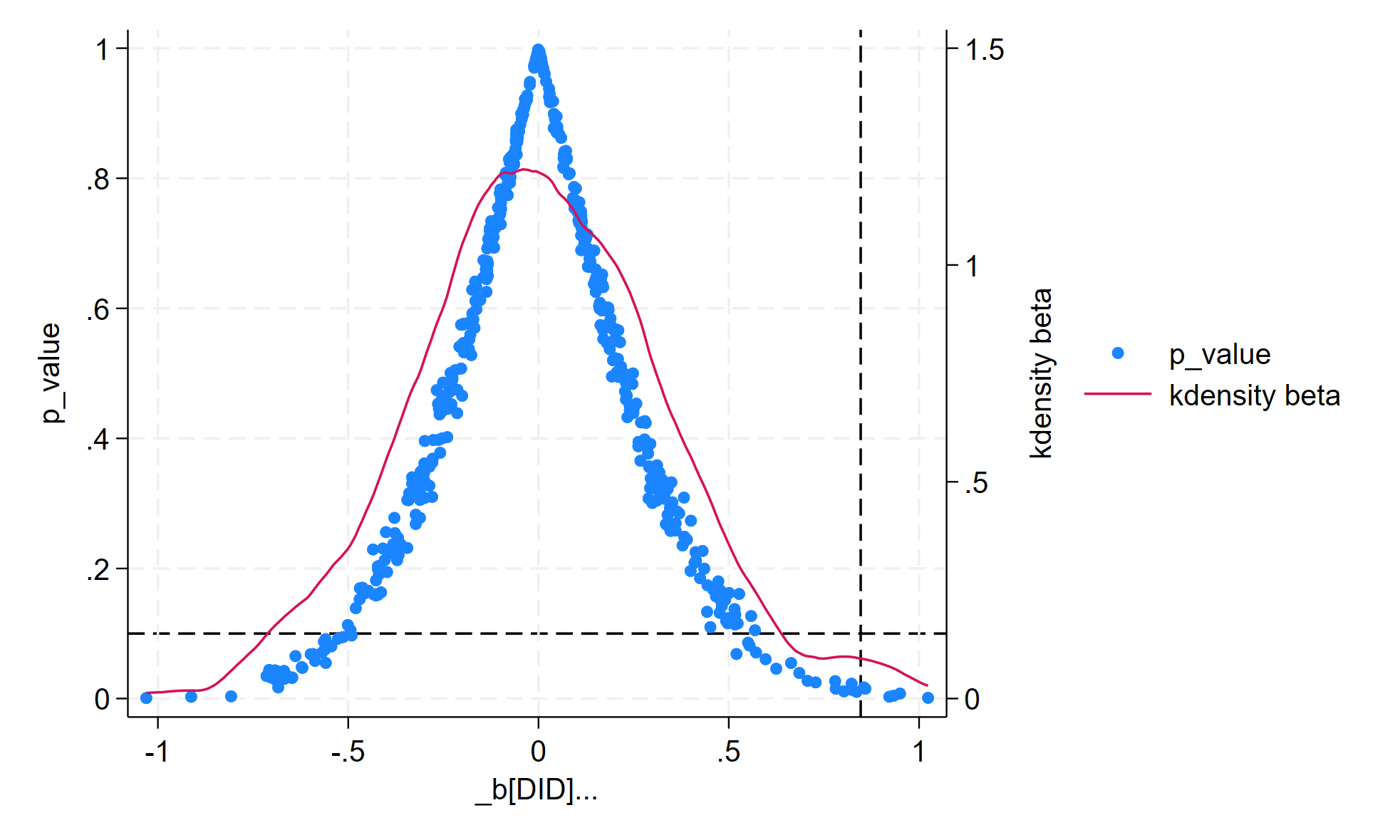

Supplement: Supplemental Material [file JPPP_A_2680235_SM6788.zip › Supplemental material/Appendix_Figure_A1._Placebo_test_results_b_Output.tif]

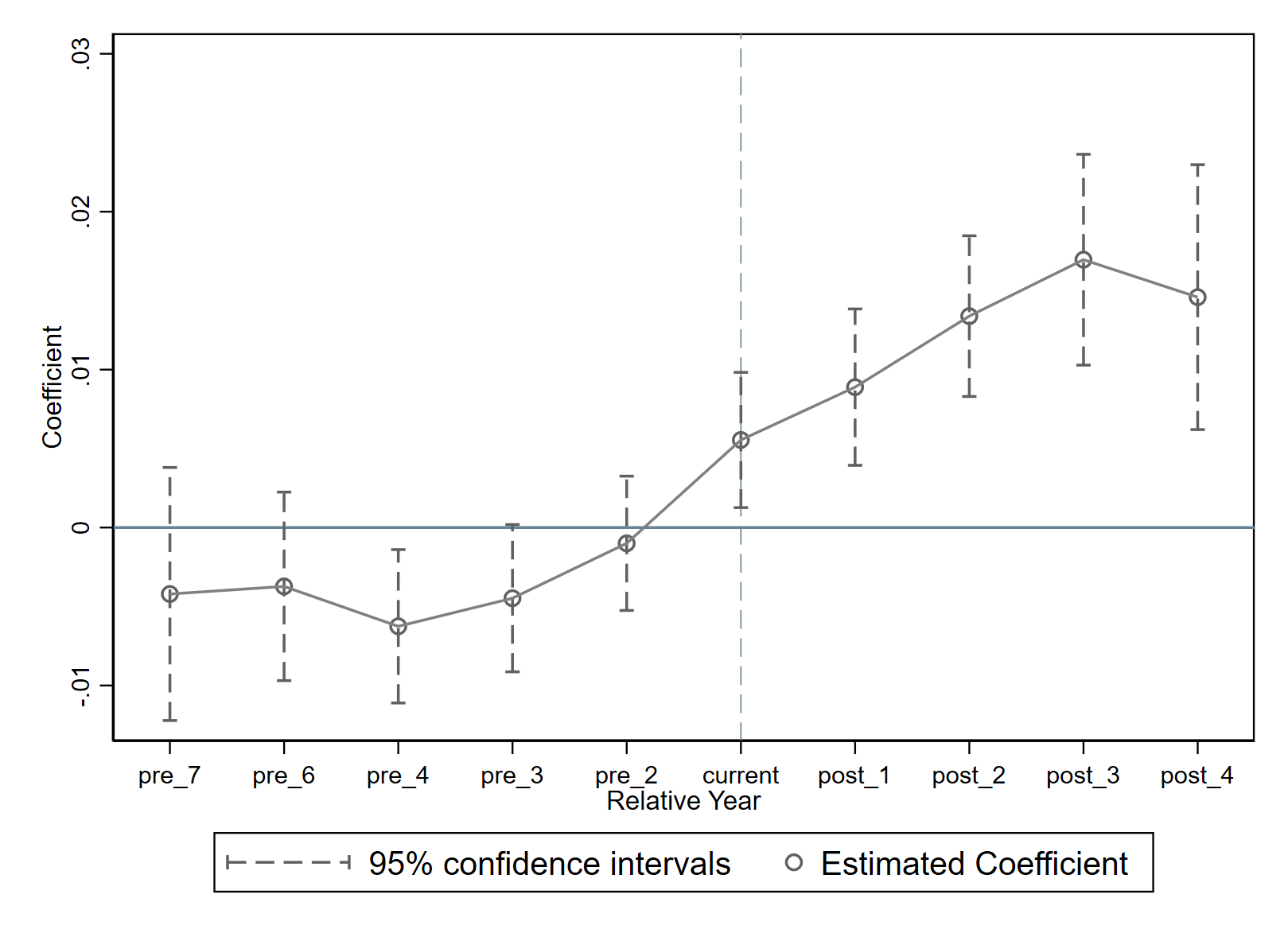

Supplement: Supplemental Material [file JPPP_A_2680235_SM6788.zip › Supplemental material/Appendix_Figure_A2._Parallel_trend_test_results_a_Input.tif]

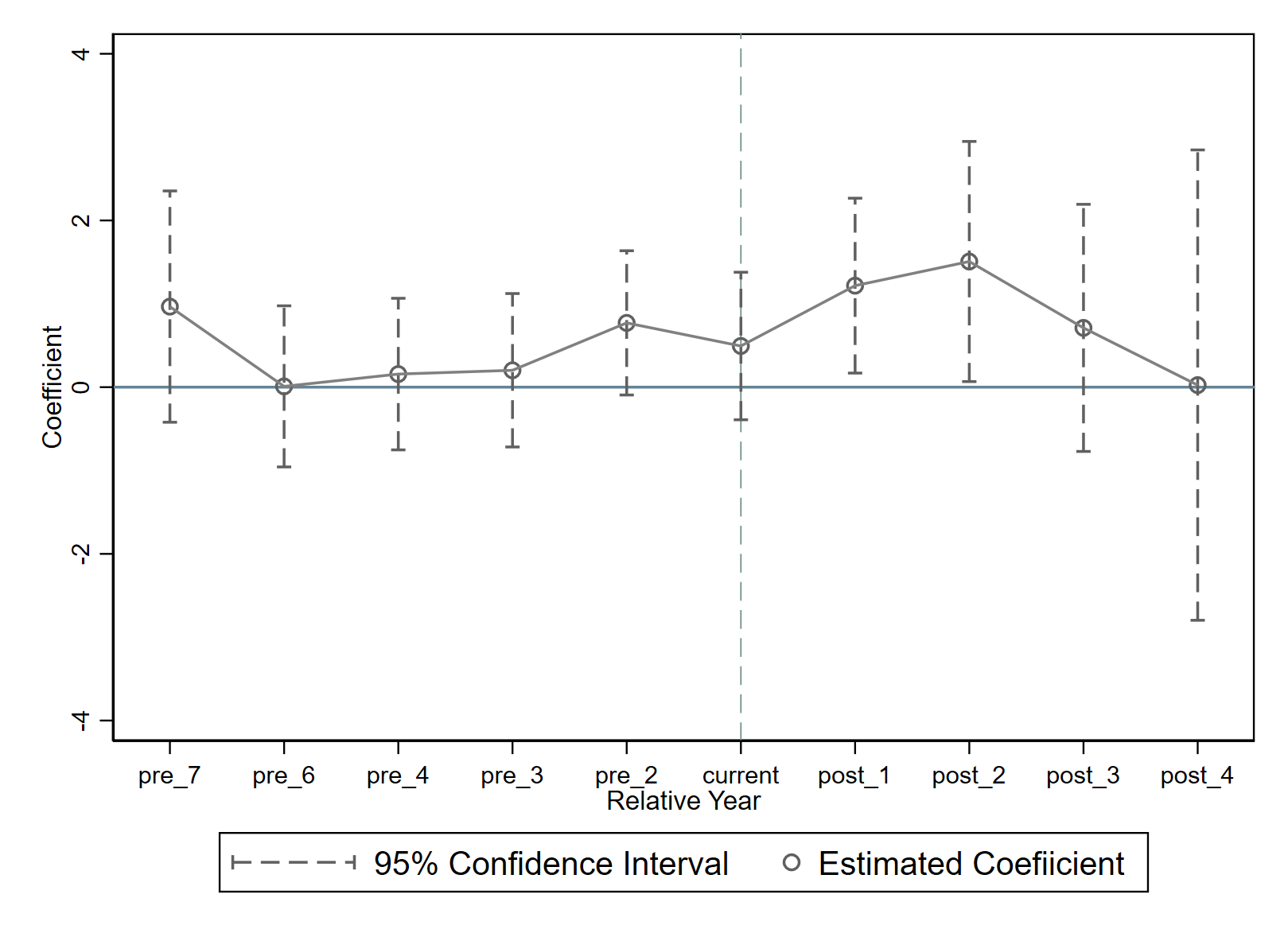

Supplement: Supplemental Material [file JPPP_A_2680235_SM6788.zip › Supplemental material/Appendix_Figure_A2._Parallel_trend_test_results_b_Output.tif]
